# Multi-omic characterization of a soil microbial consortium reveals critical role of succinate and glutamate metabolism during calcium carbonate precipitation

**DOI:** 10.1186/s12896-026-01136-y

**Published:** 2026-06-23

**Authors:** Winston E. Anthony, Tesia T. Lin, Marci Garcia, Izabel Stohel, Sharon Zhao, Natalie Sadler, Yulia Farris, Josie Eder, Sneha Couvillion, Ryan McClure

**Affiliations:** Biological Sciences Division, Pacific Northwest National Laboratory, Richland, WA USA

**Keywords:** Soil Microbiology, Microbial consortia, Microbially induced calcium carbonate precipitation, Transcriptomics, Metabolomics

## Abstract

Microbially induced calcium carbonate precipitation (MICP) holds potential for soil stabilization and carbon sequestration efforts, with the overall efficiency of the process being a major determinant for its use in many environmental and civil engineering applications. While the biogeochemical pathways and enzymes driving MICP are known, the microbial metabolic networks and community dynamics underlying such processes remain poorly characterized. To address this gap, we interrogated a MICP-capable four-member consortium of soil bacteria (*Curtobacterium flaccumfaciens*, *Rhodococcus qingshengii*, *Bacillus toyonensis* and a *Microbacterium* species), termed carbon storing consortium - A (CSC-A). Prior work shows that CSC-A yields carbonate at a higher quantity compared to the sum of carbonate individually produced by each member, suggesting MICP is driven by community dynamics. To that end we applied a multi-omic integration approach of genomics, transcriptomics, and metabolomics to investigate potential inter-species interactions that may influence the MICP phenotype. Genomic life history characterizations identified evidence of specialization by *B. toyonensis* and *Microbacterium*, while metatranscriptomic perturbation was almost ten times greater in the absence of *R. qinshengii* than *C.flaccumfaciens*, suggesting that *R. qingshengii* is a keystone species when grown in urea, a molecule key to the MICP process. By comparing individual species’ metabolomes to the metabolic profile of a shared well, we identified over 200 metabolites predicted to be produced or consumed by CSC-A members. Integrating both data types and mapping them to the KEGG reactome highlighted over 20 different enriched pathways with reactions related to glutamate metabolism, succinate metabolism, and branched chain amino acid biosynthesis. As succinate metabolism was a major node in this network we applied laboratory assays to confirm that additional added succinate led to increased carbonate precipitation by CSC-A, a critical validation of our modeling approach. By isolating and identifying the interconnected metabolic components underlying MICP in CSC-A, we identified keystone taxa, metabolites, and pathways important for future optimization of the application of this consortium to carbonate precipitation.

**Supplementary Information:**

The online version contains supplementary material available at 10.1186/s12896-026-01136-y.

## Background

A variety of microbes with microbially induced calcium carbonate precipitation (MICP) abilities have been studied for their roles in natural and engineering applications, with research rapidly gaining momentum over the past decade [1, 2]. Such applications include bioremediation, soil stabilization, and carbon sequestration [3–5]. A ubiquitous, naturally occurring process in bacterial communities, the biomineralization and precipitation of calcium carbonate (CaCO_3_) is a byproduct of microbial metabolism. However, some of the exact roles and dynamics between bacterial species that drive this precipitation are still unclear [1]. Environmental conditions (pH, moisture), urea and calcium concentrations, degradation byproducts, and ion charges all potentially influence biomineralization [4, 5] and may be factors in dynamics driving community production of carbonate. Some research has already identified specific metabolic activities and community interactions that enhance the mineralization processes [1], increasing ‘passive’ induction given the right external conditions [1, 6].

One such metabolic pathway of interest for MICP is the urease-assisted degradation of urea into ammonia and carbonic acid [7]. The equilibration of these products increases environmental pH levels, leading to the formation of carbonate ions and, given sufficient ^Ca2+^ availability in the soil, the precipitation of CaCO_3_ [8, 9]. Several studies have found that increasing the pH of environmental solutions results in faster activation of ureolytic microorganisms, simultaneously increasing CaCO_3_ precipitation [10, 11]. This requires participating microbes to maintain lower, internal pH levels, striving to achieve a state of equilibrium among byproducts, CaCO_3_, and ammonium concentrations.

More recently, pathways driven by carbonic anhydrase (CA) have also been of interest for promoting and optimizing biomineralization [6, 12]; one case study suggested that *Sporosarcina pasteurii* uses this enzyme to help reverse dehydration to maintain cell homeostasis under urea-rich conditions [13]. In most other studies, the hydrolyzing of CO_2_ by CA is crucial to maximizing CO_2_ sequestration; thus, the enzyme is typically highlighted when manipulating MICP systems [14]. However, MICP production by urease enzymes is still the most energy efficient and common way to produce carbonate.

Studies have also begun focusing on investigation of multi-species communities of MICP microbes with the goal of improving the resilience of such consortia, developing bioproduction systems, or maximizing carbon storing capacities [5, 10, 15]. Typically, MICP research studies favored modeling single-microbe systems in isolated environmental applications using *Bacillus* spp., *Sporosarcina* spp., and occasionally *Desulfovibrio* spp [1, 5, 13, 15]. Where there has been some research on curated communities of ureolytic and nonureolytic bacterial strains, these studies have yielded positive initial results for increasing CaCO_3_ production [6, 16–18]. Yet few studies have been conducted on naturally-derived consortia, and even fewer attempt to elucidate the metabolic interactions occurring among and between consortia members that lead to carbonate produciton [11, 17].

To identify an economically viable MICP biosystem, we isolated a naturally derived, four-member consortium of soil bacteria capable of MICP, referred to as Carbon Storing Consortium A (CSC-A), collected from the Kearney Research and Extension Center near Davis, CA. From this community, we aimed to characterize the metabolic potential of each microbial member, identify their respective functional niche in a complex MICP system, and map potential molecular interactions that may be key to MICP processes. Through the integration of multiple ‘omics methods, we find evidence that the 4-member community encodes for diverse functional potentials in their genomes, and expression analyses confirm that removing individual species has markedly different effects on the metatranscriptome. Integrating transcriptomic and metabolomic data identified a set of cross-pathway metabolic reactions, centered around glutamate and glutamine as keystone metabolites, that drive a community-wide response linked to amino acid metabolism during MICP. We also confirmed that other interaction predictions made by the model, centered on succinate, led to strategies to modify growth of CSC-A and enhanced carbonate production. These interactions ultimately allow the consortia to degrade urea more efficiently than just a single species, resulting in the previously observed phenotype of enhanced calcium carbonate production in the consortium. Our cross-sectional exploration culminated in the identification of species-specific roles in urea-degradation and stress-tolerance, which are important for enhancing MICP processes, laying a foundation for manipulation of metabolic exchanges in future studies to enhance and harness the capabilities of this and other naturally derived carbon sequestering communities.

## Methods

### Bacterial cell culture

All CSC-A constituents were revived from freezer stocks (stored with 25% glycerol at -80 °C) and cultured on low-nutrient Reasoner’s 2 A (R2A) agar plates for 48 h at 20 °C. Growth assays were performed in 20 °C environments and agitated at 150 rpm in a New Brunswick 2180 shaker. Species were cultivated according to the needs of the experiment (described below) in media comprised of 4 g/L of yeast extract and 5 g/L of dextrose enriched with 20 g/L of calcium chloride (5% concentration of total volume) with DI water adjusted to 1 L (B4 media), or media comprised of 4 g/L of yeast extract and 5 g/L of dextrose enriched with 20 g/L of calcium chloride and 20 g/L of urea (B4UZN media).

### Genome sequencing and analysis

CSC-A isolates were cultured in R2A media for 72 h while agitated at 180 rpm on a New Brunswick 2180 shaker at 20 °C. DNA was extracted using Zymo’s *Quick*-DNA/RNA Miniprep Kit (D7003) and sent for sequencing and assembly to Plasmidsaurus (San Francisco, CA). Assemblies were annotated using Bakta [19], and life history traits were estimated using Microtrait [20] with default parameters. Potential cross-feeding interactions between community members was estimated by generating genome scale models from genome assemblies using CarveMe [21] and then estimating interactions in minimal and M9 media using SMETANA [22]. Life-history trait analysis was performed in microtrait using their internal sequence data standardization techniques. The trait matrix was centered and scaled using the scale() R function for visualization. KEGG ortholog assignment was conducted from translated protein sequences using KofamKOALA and KOfamscan [23]. Heatmaps of traits were created using pheatmap v1.0.12 in R v4.5.0 [24, 25].

### RNA expression and profiling

CSC-A’s four microbes were cultured in two experiments. The first experiment had a total of four conditions to assess the effect of urea-enriched media: CSC-A grown in a B4 medium (composed of dextrose, yeast, and calcium chloride), CSC-A without *C. flaccumfaciens* grown in B4 medium, CSC-A grown in urea-enriched B4 (B4UZN, B4 with the addition of 2% urea), and CSC-A without *C. flaccumfaciens* grown in B4UZN. Cultures were started as above on agar plates before being moved to liquid cultivation in glass flasks. Each condition had three replicate flasks of 60 mL of its respective media, incubated at 20 °C with samples drawn at timepoints described below. For each sample 7 mL of media was removed and allowed to sit for 10 min at room temperature, to allow for precipitated CaCO_3_ to settle to bottom of tube. Supernatants were then removed from this tube and centrifuged at 5000 g for five minutes, and the centrifugation supernatant removed. The pellets for each replicate were then stored in a -80 °C freezer until extraction. Timepoints collected for this first experiment were 0, 24 and 48 h. This setup is repeated for the second experiment, which excludes *R. qingshengii* from the CSC-A community rather than *C. flaccumfaciens*. Timepoints for the second experiment were 24 and 48 h.

RNA extraction was performed using Zymo’s *Quick*-DNA/RNA Miniprep Kit (D7003) and sequenced by Azenta (South Plainfield, NJ) using Illumina technology. Bioconductor’s DESeq2 package (3.9) [26] was used to analyze gene expression for each member of the CSC-A community under the different conditions tested (in the presence and absence of *C. flaccumfaciens* and *R. qingshengii* or in different media types). Raw transcript counts were normalized using the internal median of ratios normalization used by DESeq2 for differential expression. The set over-enrichment and GSEA of KEGG pathways were performed on variance stabilizing transformation (vst) transformed counts generated using DESeq2, and multiple hypothesis correction was performed using the false discovery rate (fdr) method. The likelihood ratio test was used to determine changes to gene expression across timepoints for media with and without urea. Given the large numbers of genes after analysis, we had the luxury of selecting more conservative constraints for interpreting the statistical significance of our data, ultimately leading to cutoff values of a log2 fold change of < -3 or > 3 and a p-adjusted value of < 0.05 used to consider a gene differentially expressed (DE). These are the genes discussed in Tables 2 and 3. Figure 5 was plotted using ggplot2. The gseKEGG from the clusterProfiler R package was used for set over-enrichment and GSEA analysis of KEGG pathways and modules.

### Metabolomics

Each member of CSC-A was individually cultured in B4UZN media and inoculated in designated wells of a 96-well plate with filter bottoms on each well. A total of 300 uL of each species, at an O.D._600_ of 0.1, was added to each well. There were ten repeat wells for each member of CSC-A. This plate was then placed in a square reservoir pool with the filter bottoms of the wells submerged a few millimeters in the reservoir pool which contained B4UZN media, an approach similar to what has been described before [27]. Plates were incubated at 20 °C for 72 h before 250 uL was collected from each well. Samples from the same species were combined in pairs so that we ended with 5 biological replicates for each species. Samples were then spun down at 5000 g for five minutes and supernatants were used for metabolomics analysis. Samples were processed for global metabolomics using an LC-MS on a Q-Exactive HFX. Metabolite identification for each biological replicate was performed using the Sonnenburg library and a compilation of public MS/MS databases. We deployed the Omu R package [28] to process our metabolomics count data and assess each bacterial genome for orthology to protein sequences within the KEGG Orthology (KO) database [29]. Metabolite data was grouped by replicates, square-root transformed, and Welch’s t-test was used to determine statistical significance at a p-adjusted value of less than or equal to 0.01 contrasted to the control for generating the PCA of highly variable metabolites, and 0.05 when contrasted to the shared reservoir well and for identifying reactions with transcriptomic and metabolomic evidence.

### Integrated ‘Omics analysis

DE transcripts were first generated using DESeq2 in R from the metatranscriptome that mapped to KEGG orthologs using KOfamscan using a threshold of p-adjusted < 0.05. Orthologs with multiple hits were screened by keeping the locus with the highest average base mean. All reactions associated with DE KEGG orthologs and DE KEGG metabolites were queried from the KEGG REST API [30] using the KeggREST v1.48.0 [31] package in R. Reaction subnetworks with DE in both data types were extracted from the relevant KEGG Pathway graphs using ggkegg [32] and visualized in R.

## Results

### CSC-A is a genetically diverse soil microbial consortium with each member encoding unique stress tolerance traits and niche adaptations

CSC-A is derived from naturally co-occurring bacteria sourced from an agricultural setting in Davis, California. The community is made of four members, which were separately isolated, cultured, and sequenced in the laboratory. Reads were de novo assembled to identify their taxonomic assignment and estimate their functional and metabolic potential. Development of this community and other measurements, such as pH and organic carbon levels during growth, are detailed in a previous publication (https://pubmed.ncbi.nlm.nih.gov/41432929/). Our prior publication also contains X-ray diffraction analysis of the CaCO_3_, confirming that it is composed of 75% vaterite and 5% calcite. The remaining 20% is amorphous and cannot be identified.

Three of the four community members were identified to the species level: *Rhodococcus qingshengii* and *Curtobacterium flaccumfaciens* from the Actinomycetota phylum and *Bacillus toyonensis* from the Bacillota phylum. One member was identified to the genus *Microbacterium* within the phylum Actinomycetota; however, this isolate fell outside the 95% average nucleotide identity threshold for assignment to any of the type strains within the genus.

*R. qingshengii* exhibited the largest genome at 7.2 Mb, followed by *B. toyonensis* at 5.3 Mb. The *Microbacterium sp.* (hereafter referred to as *Microbacterium*) and *C. flaccumfaciens* both had smaller genomes at 3.9 and 3.8 Mb, respectively (Table S1). *C. flaccumfaciens* was estimated to have the longest minimum generation time (MGT), (2.1 h^− 1^) and the highest optimum growth temperature (OGT) and GC content at 34.1 °C and 71%, respectively. In order of decreasing MGT, *Microbacterium*, *R. qingshengii*, and *B. toyonensis* had estimated MGTs of 1.2, 1.2, and 0.4 (h^− 1^), respectively. The community also exhibited a wide OGT range, with *B. toyonensis*, *Microbacterium*, and *R. qingshengii* estimated to grow best at 31.9 °C, 31.8 °C, and 24.3 °C (temperatures routine to the soil from which these species were isolated), respectively. Thus, CSC-A contains a diverse set of species with a range of genetic characteristics and growth dynamics.

**Fig. 1:**
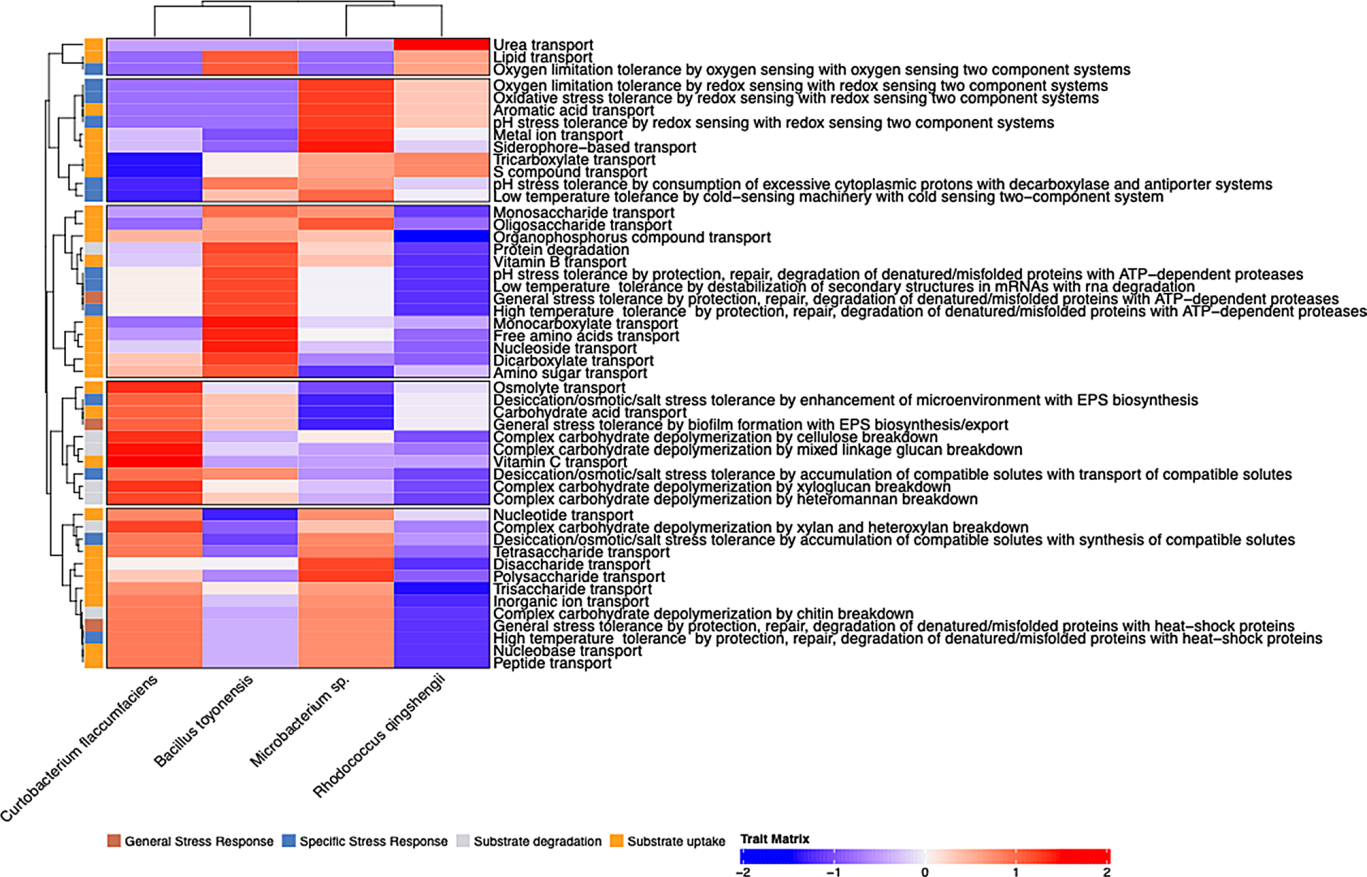
Heat map of predicted functional potential of each CSC-A community member. Each row represents the functional enrichment or depletion of a life-history trait. Rows are split into 5 groups based off of hierarchical clustering. Red cells indicate enrichment of a trait in an organism and blue indicates depletion. Values are trait counts normalized to genome length. The bar on the left side of the heatmap indicates which category of life-history trait each row corresponds to

To interrogate community dynamics, characterization of the functional potential of the community was performed by mapping translated protein sequences from each member to a unique profile of functional trait representations (Fig. 1). We also conducted KEGG ortholog analyses to map genes to known metabolic pathways and reactions. Though there is evidence in literature that some species of *Microbacterium* and *B. toyonensis* possess urease [33–35], both the functional trait representation and ortholog mapping identified *R. qingshengii* as the only member with a genome containing the urea transport and urease system. Both *R. qingshengii* and *C. flaccumfaciens* possessed the urea carboxylase and allophanate hydrolase enzymes necessary for urea amidolyase dependent degradation of urea in our systems. *Microbacterium* had some of the highest trait representation for resource scavenging, such as the transport of aromatic compounds, various polysaccharides, as well as small molecules such as metal ions, sulfur, and siderophores. While *Microbacterium* primarily exhibited stress response traits assigned to two-component systems, *B. toyonensis* instead carried a relatively large genetic complement of stress mitigation (general, heat, cold, and pH) through ATP−dependent proteases. *C. flaccumfaciens* exhibited the unique ability to transport and depolymerize multiple complex carbohydrates: xylan, heteromannan, cellulose, and mixed-linkage glucans. *C. flaccumfaciens* also possesses genes increasing tolerance to osmotic and generalized stress through bacterial exopolysaccharide (EPS) biosynthesis as well as biofilm formation. Finally, *C. flaccumfaciens* and *Microbacterium* both shared a set of traits related to saccharide transport, stress tolerance (via heat-shock proteins), and the ability to degrade chitin. Given the varied functional potential, we anticipated a large degree of specialization during co-culture, and that removing individual members would significantly perturb community metabolic output.

### *R. qingshengii* loss results in a larger perturbation to the metatranscriptome compared to *C. flaccumfaciens*

**Fig. 2:**
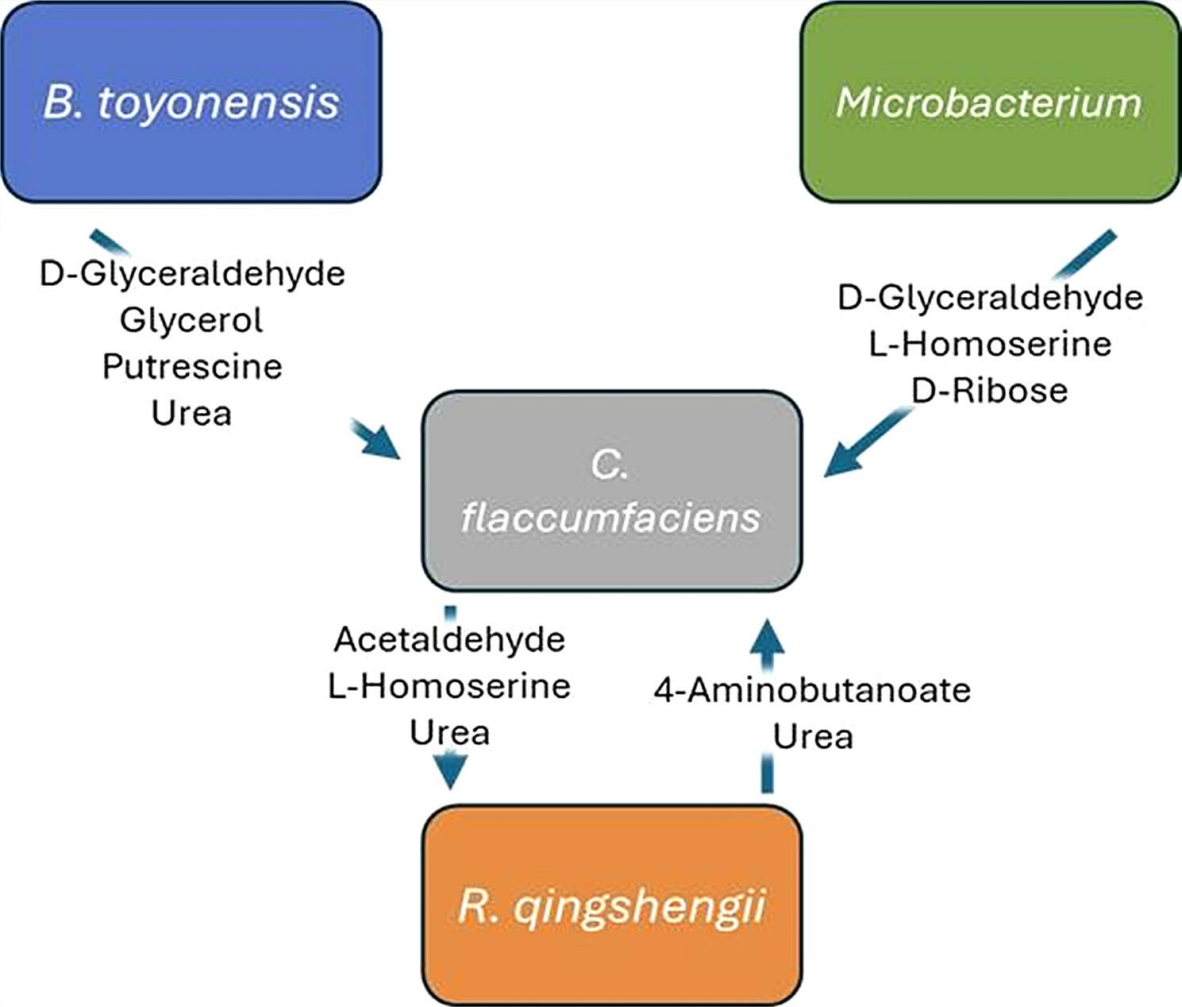
Preliminary predictive modeling of metabolic exchange reactions that could be occurring between the members of CSC-A

Considering the unique metabolic potential found in each member of CSC-A, we sought to quantify the impact removing specific community members would have on the metatranscriptome during growth with urea. An initial metabolic exchange model generated using draft genome scale models alongside outcomes from Bakta and Smetana mapped the unique potential of each species; it ultimately suggested that most exchange interactions were centered around *C. flaccumfaciens.* The exchange model (Fig. 2) predicts it to serve as a receiver for metabolites from all other consortia members, while simultaneously acting as a metabolite donor for *R. qingshengii*. We mapped KEGG ontology ID’s to a total of 10,368 loci in the CSC-A concatenated set of genomes. *R. qingshengii* was identified as the only species with a KEGG ortholog (KO) for the urea transporter and urease system and the urea amidolyase enzyme (Fig. 1). Additionally, *C. flaccumfaciens* is the only species (other than *R. qingshengii*) that possesses orthologs for urea amidolyase. Given the genetic evidence of urea degradation in these two community members, and the likelihood that they participated in exchange of urea and other metabolites between each other and the rest of CSC-A, we perturbed the community structure through removing each of these members one at a time in iterative experiments and in media with and without urea for a better understanding of how urea relates to the role of these species in the community.

**Fig. 3:**
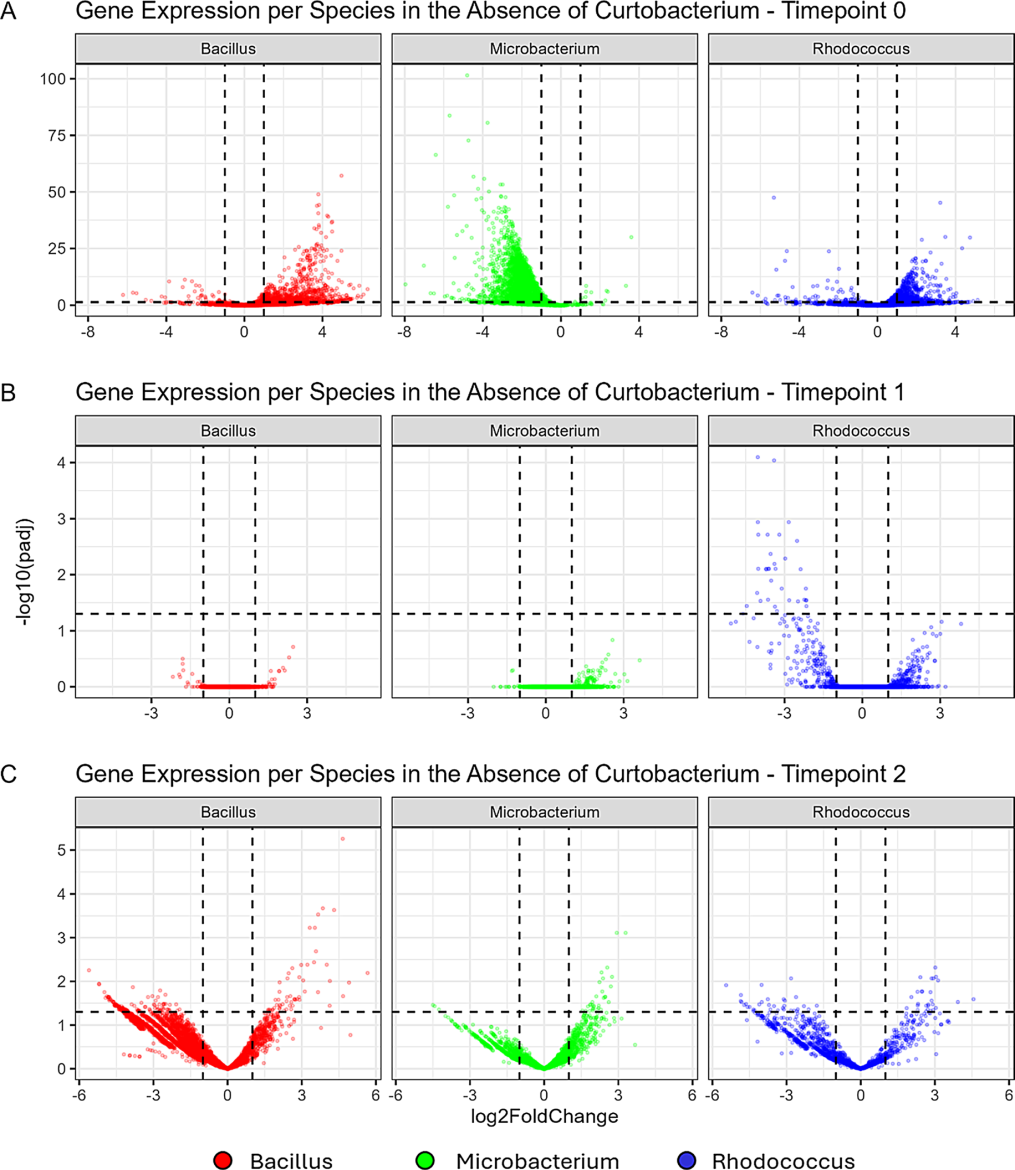
Differential gene expressions of species (*Bacillus toyonensis*,* Microbacterium spp*, and *Rhodococcus qingshengii*) in CSC-A with and without *Curtobacterium flaccumfaciens*. Volcano plot values were selected for a p-adj value of < 0.05, and logfold changes of > 3 and < − 3, then plotted throughout different time points (Timepoint 0 = 0 h (**A**), Timepoint 1 = 24 h (**B**), Timepoint 2 = 48 h (**C**))

When comparing communities with and without *C. flaccumfaciens* (Locus ID prefix IHGJAF), we identified a total of 885 highly differentially expressed genes (DEGs) (-3 < Log2FoldChange > 3 and an adjusted *p-*value of < 0.05), (Fig. 3; Table 1) across a 48-hour time course (0 – inoculation, 24, and 48 h). We did not recover evidence of any transcriptional changes during removal of *C. flaccumfaciens* directly relating to urea or MICP related metabolic functions suggesting that *C. flaccumfaciens* may not be heavily involved in these specific processes. We observed no significant gene upregulation in the 24-hour sample, with all DEGs exhibiting decreased expression as a function of *C. flaccumfaciens* loss and mapping to the *R. qingshengii* (Locus ID prefix KXIUOE) transcriptome. Of these, statistically significant DEGs coded for transferases like the antigen biosynthesis glycosyltransferase and degradation enzymes for valine, leucine, threonine, and isoleucine and CA. Across the 24- and 48-hour timepoints, other significant DEGs coded for catalytic enzymes, and transport proteins (Table 2). At the 48-hour time point, genes expressed at higher levels in the absence of *C. flaccumfaciens* were mostly associated with *B. toyonensis* and mapped to functions such as maintaining cell survival, metabolic regulation, phosphotransferase enzymes and other degradation catalysts. Other significant DEGs at 48 h were mapped to ribosomes and DNA forming proteins in *B.toyonensis*, *Microbacterium* and *R. qingshengii* (Table 2). Fold change values for all DE genes are shown in Table S2. Normalized expression levels for all genes are shown in Table S3 and metadata information on sample names is shown in Table S4.

**Table 1:** At a cutoff of Log2Fold Change of ≥ 3 and ≤-3, with a p-adjusted value of ≤ 0.05, this table represents the relative number of genes contributed from each of CSC-A’s four members, under two conditions tested: CSC-A without *C. flaccumfaciens*, and CSC-A without *R*. *qingshengii.* Cells with ‘NA’ indicate that there were no samples available at that specific time point, or from a specific consortia member

Number of DEGs Responding to Loss of CSC-A Members							
		No *C. flaccumfaciens*			No *R. qingshengii*		
Species	Gene Count	0 h	24 h	48 h	0 h	24 h	48 h
*B. toyonensis*	5972	(+) 364	0	(+) 18	NA	(+)5227	(+)5491
		(-) 13	0	(-) 110	NA	0	0
*Microbacterium*	3751	(+) 2	0	(+) 1	NA	0	(+) 9
		(-) 256	0	(-) 3	NA	(-) 755	(-) 1
*R. qingshengii*	6625	(+) 46	0	(+) 6	NA	NA	NA
		(-) 34	(-) 23	(-) 24	NA	NA	NA
*C. flaccumfaciens*	3578	NA	NA	NA	NA	(+) 231	(+)2492
		NA	NA	NA	NA	(-) 50	0

**Table 2:** Highly differentially expressed genes (DEG) in the absence of *C. flaccumfaciens*. Log2foldchange refers to comparisons between CSC-A with *C. flaccumfaciens* and CSC-A without *C. flaccumfaciens*. All gene expression levels for all samples are shown in Supplementary Table 3

Highly Regulated Genes Responding to Loss of *C. flaccumfaciens*			
Time Point	Locus_ID	Kegg Orthology Annotation	Log2FoldChange
24 h	KXIUOE_10170	3-oxoacid CoA-transferase subunit A [EC:2.8.3.5]	-4.45667
24 h	KXIUOE_10175	citrate lyase subunit beta / citryl-CoA lyase	-4.0291
24 h	KXIUOE_26600	ABC-2 type transport system permease protein	-3.63777
24 h	KXIUOE_09545	threonine dehydratase [EC:4.3.1.19]	-3.59961
24 h	KXIUOE_09560	NAD(P)H dehydrogenase (quinone) [EC:1.6.5.2]	-3.5381
48 h	HBOIFM_26945	small subunit ribosomal protein S14	4.910015
48 h	HBOIFM_27020	elongation factor Tu	4.668582
48 h	HBOIFM_01850	enolase 1/2/3 [EC:4.2.1.11]	4.136212
48 h	HBOIFM_26985	large subunit ribosomal protein L22	4.009114
48 h	KXIUOE_20195	single-strand DNA-binding protein	3.929714
48 h	HBOIFM_07195	phosphoenolpyruvate-protein phosphotransferase (PTS system enzyme I)	3.57345
48 h	HBOIFM_22750	peptide/nickel transport system ATP-binding protein	-4.74237
48 h	HBOIFM_06015	glucokinase	-4.77976
48 h	HBOIFM_01520	UDP-N-acetylglucosamine 2-epimerase (non-hydrolysing)	-4.81036
48 h	HBOIFM_04555	CAAX protease family protein	-4.814
48 h	KXIUOE_32995	DNA repair protein RecN (Recombination protein N)	-4.81987
48 h	HBOIFM_30080	putative transposase	-4.83498
48 h	KXIUOE_26600	ABC-2 type transport system permease protein	-4.84472
48 h	HBOIFM_03735	GTP 3’,8-cyclase [EC:4.1.99.22]	-4.88415
48 h	HBOIFM_21135	ribosomal protein S12 methylthiotransferase accessory factor	-5.19512
48 h	KXIUOE_07305	peptide-methionine (R)-S-oxide reductase	-5.44289
48 h	HBOIFM_02175	methyl-accepting chemotaxis protein	-5.60861

**Table 3:** Highly differentially expressed genes (DEG) in the absence of *R. qingshengii*. Log2foldchange refers to comparisons between CSC-A with *R. qingshengii* and CSC-A without *R. qingshengii*. All gene expression levels for all samples are shown in Supplementary Figure X

Highly Regulated Genes Responding to Loss of *R. qingshengii*			
Time Point	Locus_ID	Kegg Orthology Annotation	Log2FoldChange
24 h	HBOIFM_01930	carbonic anhydrase	17.51671
24 h	HBOIFM_01925	cyanate lyase	16.30717
24 h	HBOIFM_10890	regulatory protein spx	15.40686
24 h	HBOIFM_04790	thioredoxin	15.30231
24 h	HBOIFM_15415	dipeptidase D	14.88725
24 h	HBOIFM_02680	iron-sulfur cluster assembly protein	14.57988
24 h	HBOIFM_10570	N-acetylglucosamine malate deacetylase 2	14.46656
24 h	HBOIFM_25530	hydroxyethylthiazole kinase	14.3682
24 h	HBOIFM_05925	zinc transport system permease protein	14.36612
24 h	PGEDEG_04040	3-oxoacid CoA-transferase subunit B	-7.00343
24 h	PGEDEG_02340	DNA polymerase III subunit delta	-7.19443
24 h	PGEDEG_06485	ring-1,2-phenylacetyl-CoA epoxidase subunit PaaA	-7.27149
24 h	PGEDEG_02345	acetyl-CoA acyltransferase	-8.69249
24 h	PGEDEG_05120	ribosome hibernation promoting factor	-8.73803
24 h	PGEDEG_04310	small subunit ribosomal protein S15	-9.95552
48 h	HBOIFM_04235	argininosuccinate synthase	14.71323
48 h	HBOIFM_06655	bacilliredoxin	14.68865
48 h	HBOIFM_10890	regulatory protein spx	14.09783
48 h	HBOIFM_10295	zinc D-Ala-D-Ala carboxypeptidase	13.96936
48 h	HBOIFM_02155	quercetin 2,3-dioxygenase	13.66819
48 h	HBOIFM_01930	carbonic anhydrase	13.6558
48 h	HBOIFM_16165	foldase protein PrsA	13.64658

**Fig. 4:**
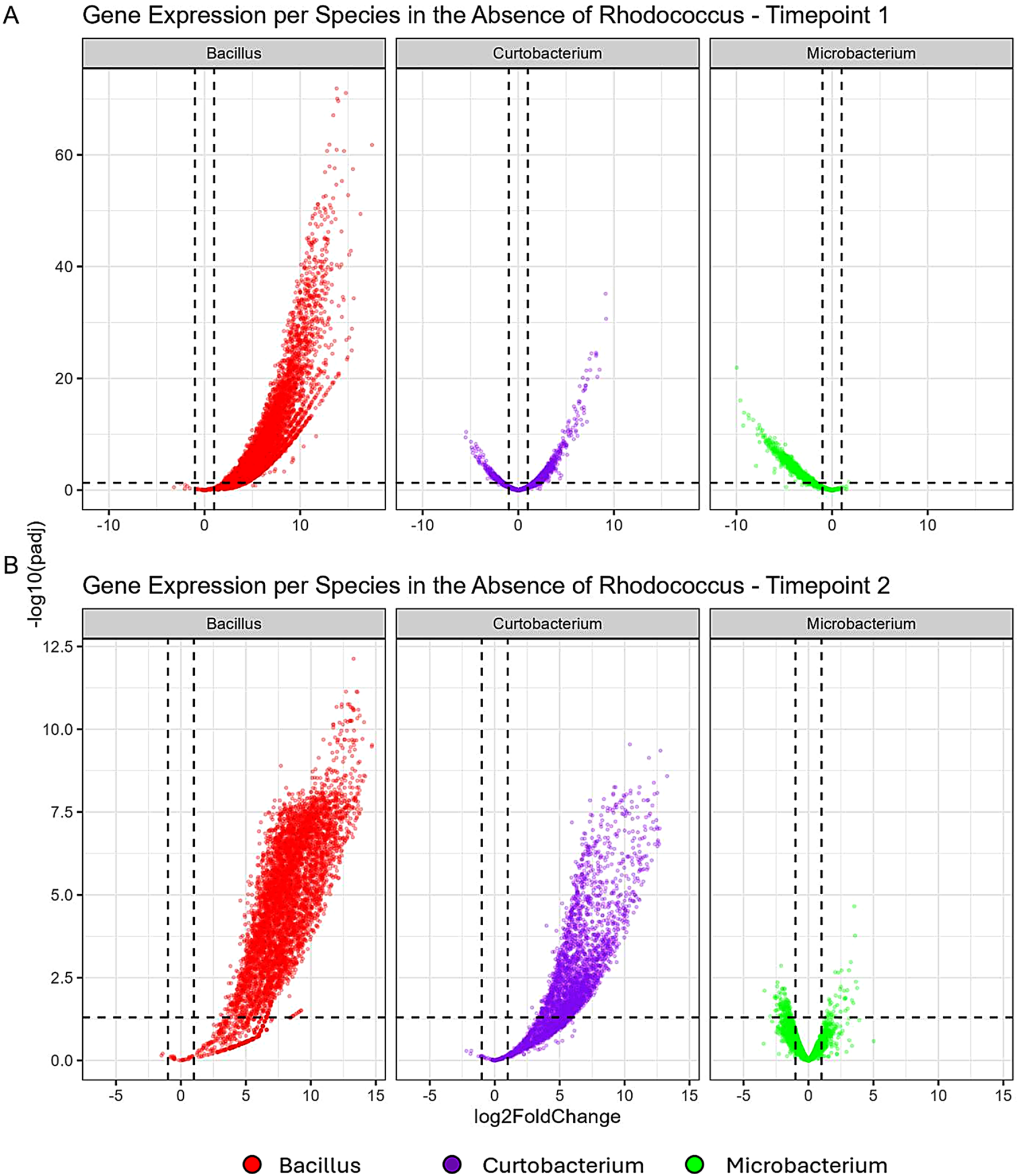
Differential gene expressions of species (*Bacillus toyonensis*, *Curtobacterium flaccumfaciens*, and *Microbacterium* spp.) in CSC-A with and without *Rhodococcus qingshengii*. Volcano plot values were selected for a p-adj value of < 0.05, and logfold changes of 3 and − 3, then plotted throughout different time points (Timepoint 1 = 24 h (**A**), Timepoint 2 = 48 h (**B**))

Alternatively, when *R. qingshengii* was removed from CSC-A and the complete consortia was contrasted with the absence of *R.qingshengii*, we identified a total of 9,009 DEGs between the 24 and 48 h timepoints —more than ten times as many genes as was identified during the absence of *C. flaccumfaciens* (Table 1). Removing *R. qingshengii* resulted in strong upregulation of *B. toyonensis* genes coding for stress, regulatory and stabilizing responses, bicarbonate conversion and CA, kinases and other catalysts, and zinc transport proteins (Fig. 4; Table 3) at the 24-hour timepoint. Statistically significant downregulated DEGs here were all associated with *Microbacterium* (Locus ID prefix PGEDEG), coding for flagellar biosynthesis and locomotion, and protein synthesis inhibition genes. At the 48-hour time point, significant DEGs all belonged to *B. toyonensis* and included thioredoxin genes, transcriptional regulators, and arginosuccinate synthase. Given the significant impact of the removal of *R. qingshengii* from the CSC-A community on ensuing gene expression, we decided to further explore the role *R. qingshengii* in the context of MICP.

**Fig. 5:**
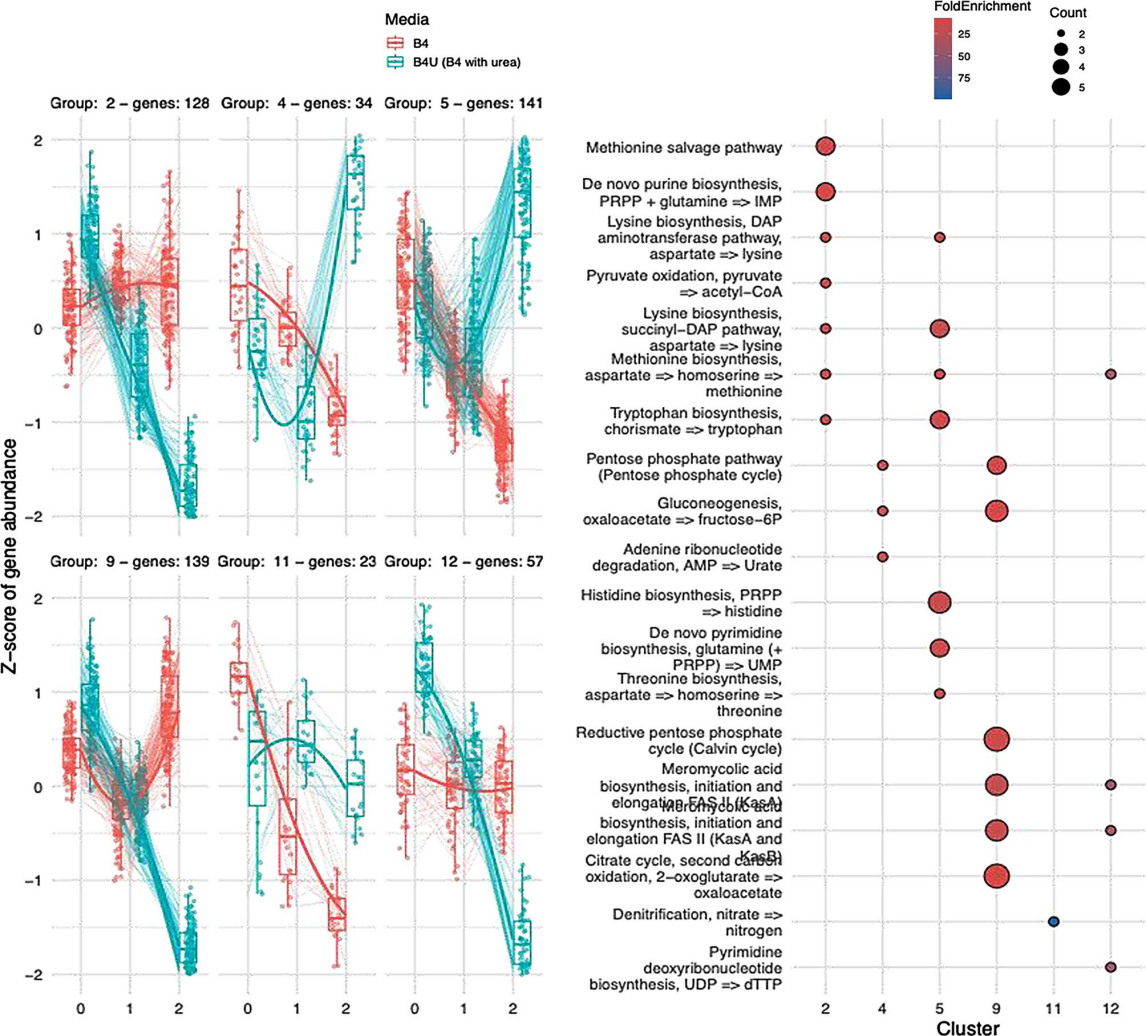
Cluster analysis of temporally co-expressed genes during growth in urea. Left: each group represents a set of co-expressed genes across three timepoints. Bold lines across time points are general additive model fits for each group across time: 0: Inoculation, 1: 24 h timepoint, and 2: 48 h timepoint. Right: Dot-plot indicating significant KEGG module set-overrepresentation for each cluster. Dot size indicates the number of genes within the gene group that belong to each module, and color indicates the enrichment value

According to our functional trait potential predictions, at least one community member, *R. qingshengii* was capable of urea degradation via hydrolysis. This process and its ensuing precipitated product are likely to result in altered metabolic requirements for growth, due to the increased pH and resulting breakdown products over time. To better understand the effects of added urea to the functional output of CSC-A, we used the likelihood ratio test, paired with cluster analysis, to identify sets of co-expressed genes exhibiting significant changes in expression over 3 sampling points (0, 24 and 48 h) using meta-transcriptomics profiles from the complete CSC-A grown in either base B4 media or B4 with urea added (B4UZN). We identified multiple clusters containing DE co-expressed genes from all community members and tested each for KEGG module enrichment. We identified 11 total clusters of significantly co-expressed genes, 6 of which contained a larger number of genes from specific KEGG modules than expected by chance (Fig. 5). 48% (1518) of the DE genes came from *R. qingshengii*, followed by *C. flaccumfaciens* 1080 (35%), *Microbacterium* 342 (11%), and finally *B. toyonensis* 192 (6%). Clusters 2 (128 genes), 9 (139 genes), and 12 (57 genes) decrease in expression over sampling points, are dominated by *R. qingshengii* genes (77%), contain amino acid metabolism modules (such as methionine salvage, de novo purine biosynthesis from glutamine), in addition to decreased expression of meromycolic acid biosynthesis and central metabolism modules such as the Calvin and Citrate cycles. Clusters 4 and 5 contained 175 genes which increased inexpression after 48 h in B4UZN media but decreased expression in control B4 media at this same timepoint. These groups contained mostly *C. flaccumfaciens* genes related to amino acid metabolism that overlapped with cluster 1’s energy modules, such as the pentose phosphate pathway and gluconeogenesis. Finally, cluster 11 contained 23 genes split relatively equally among community members related to the denitrification KEGG module that converts nitrate to nitrogen. This group exhibited no change in expression during growth in urea, but significantly decreases over time in control media.

### Taxa specific metabolomics identifies differences between community and individual metabolome profiles

We identified metabolites that were potentially consumed, precipitated, and exchanged between members of the CSC-A consortium when grown in B4UZN media. To assess this, we made use of an individual-and-shared-reservoir experimental set up where single species cultures were inoculated into individual wells, the bottoms of which were made of a semi-permeable membrane rather than plastic. Wells are then connected to each other through a shared reservoir at the bottom of the plate, allowing for metabolite and nutrient transfer while maintaining segregation of species. Metabolite samples from individual wells, when compared to the uninoculated control media, helps compile metabolic profiles for each individual species. Profiles were also compared to the shared reservoir to determine species-specific enrichment or depletion of individual metabolites.

**Fig. 6:**
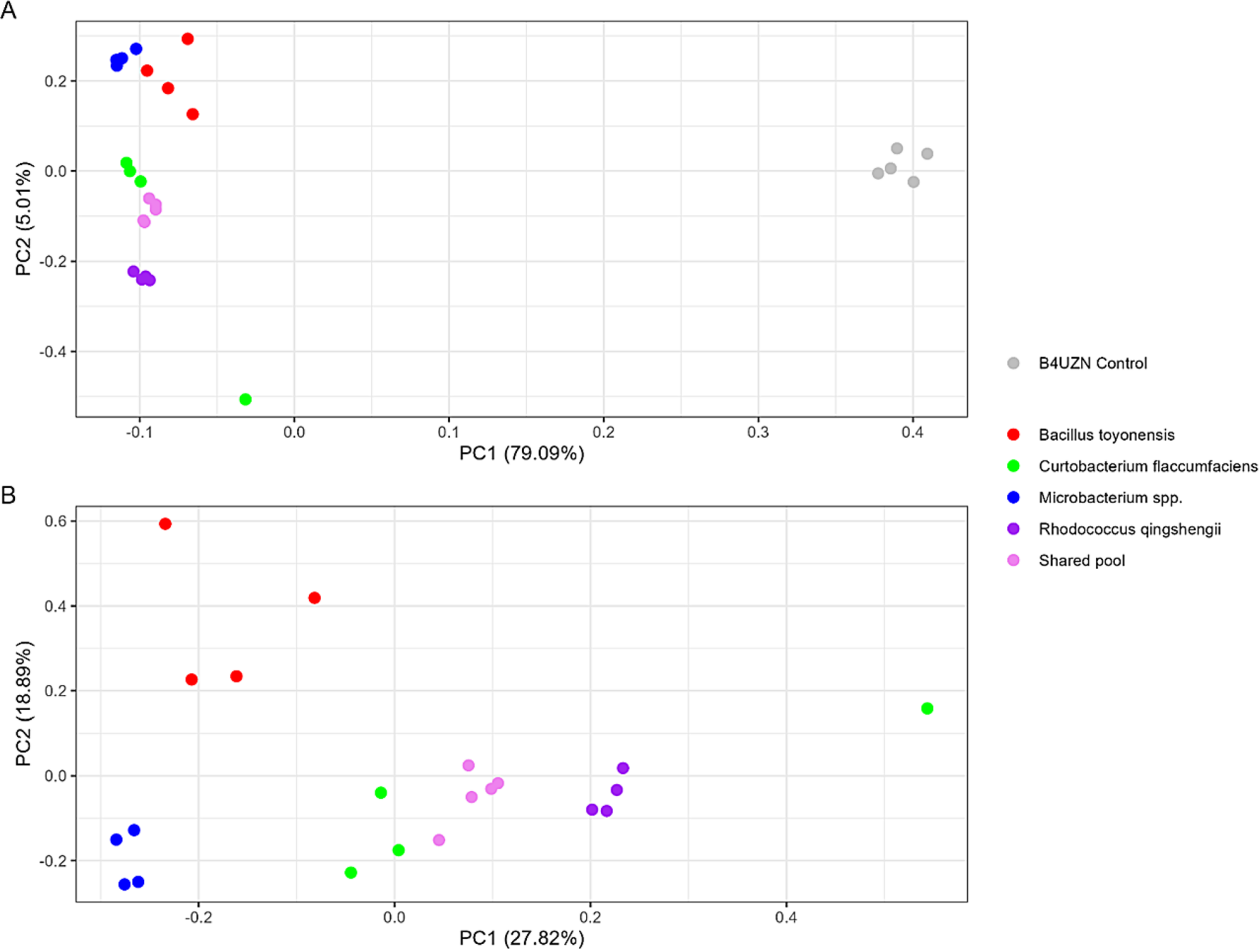
Principle Component Analysis (PCA) plot illustrating metabolite expression between CSC-A members with B4UZN control media (**A**), and without control B4UZN media (**B**) for contrast. The stark difference in variance between the control group (**A**) to other metabolite expressions (CSC-A species and shared pool) suggests a strong distinction between metabolite samples from the control group and all other groups

Principal Component Analyses comparing individual profiles to those of uninoculated control media and the shared well (Fig. 6A) indicated a significant change in metabolite composition during community growth, with the first principal component explaining 79% of the variation in the top 500 most variable metabolites. When removing the uninoculated control media from assessment, metabolic profiles from *C. flaccumfaciens* were located closest to the shared reservoir samples, followed by *R. qingshengii* and *Microbacterium* (Fig. 6B). Along the first and second principal components, *B. toyonensis* contributed the only set of profiles which were distinctly separate from the shared reservoir samples.

The top eight significantly enriched metabolites (when contrasted to a media control of B4UZN) in the shared reservoir after a five-day growth period were Camphor-10-sulfonic acid, N-(5-acetamidopentyl) acetamide, indole-3-lactic acid, 1,3-Cyclohexanedicarboxylic acid, 4-Hydroxy-6-methyl-2-pyrone, 3,5-Dichlorosalicylic acid, 4-Hydroxyphenyllactic acid, and monobutyl phthalate—most of which are likely metabolic byproducts. The top eight metabolites significantly depleted were sn-Glycero-3-phosphocholine, nicotinamide, val-thr, val-val, d-xylose, 2,3-Naphthalenedicarboxylic acid, L-Asparagine, and arginine—many of which are amino acids or amino acid dipeptides involved in the biosynthesis of proteins or organic compounds. These likely represent the metabolites that CSC-A is producing or consuming, respectively, at the community level.

**Fig. 7:**
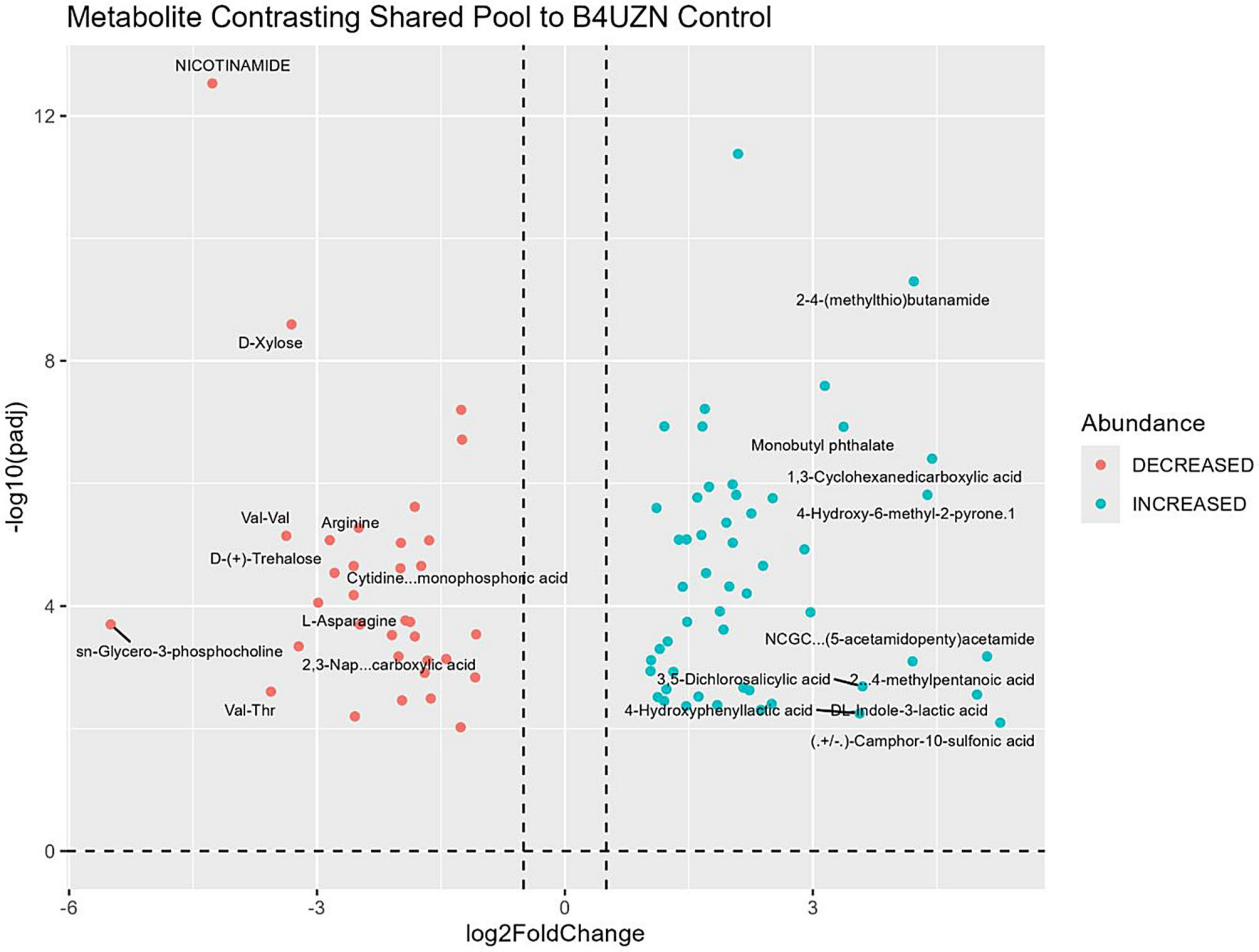
Volcano plot of metabolite expression when contrasting metabolites from the shared pool to those in the control B4UZN media

We next tested for enrichment and depletion of metabolic byproducts at the single species level by comparing metabolites in wells containing a single species to the shared reservoir or the uninoculated control media (Table 4; Fig. 7). Ubiquitous across most species-specific metabolite profiles was as the depletion of sn-Glycero-3-phosphocholine and D-(+)-Trehalose, and the enrichment of 4-Hydroxy-6-methyl-2-pyrone. Though *B. toyonensis* was the producer of most of these differentially enriched metabolites, the metabolites with the highest enrichment-Camphor-10-sulfonic acid (Log2Fold = 5.156) and 2,3-Naphthalenedicarboxylic acid (Log2Fold = 5.012) were associated with *Microbacterium*. *R. qingshengshii* wells had the highest depletion of the metabolite sn-Glycero-3-phosphocholine (Log2Fold = -5.194). While there were significant differences in enrichment between individual wells and the shared reservoir, differences were lower in magnitude than what was observed versus the uninoculated control sample; the highest enriched metabolite was the dipeptide Ile-Leu (Log2Fold = 1.805) during growth of *B. toyonensis*, and the most depleted was the demethylation byproduct and biosynthetic precursor S-Adenosyl-homocysteine (Log2Fold = -2.349) in the *Microbacterium* well. Of the 10 metabolites with the strongest enrichment across the community, 8 were within the organic acids and derivatives KEGG superclass, while 5 of the top 8 depleted metabolites belonged to the nucleosides, nucleotides, and analogues superclass, indicative of categorical DE of metabolite classes. All detected metabolites in each well are shown in Table S5.

**Table 4:** Top 5 metabolites enriched or depleted for each community member by comparison. Two comparisons were conducted: “Control”, which compares the individual well to the individual well without any bacterial culture present, and “shared” which compares the individual well to the shared reservoir

Metabolites Consumed or Produced by CSC-A Members					
Species	Contrast	Production	Metabolites	Log2Fold	*p*-adj
*B. toyonensis*	Control	Enriched	4-Hydroxy-6-methyl-2-pyrone.1	4.435	0.000
			4-Hydroxyphenyllactic acid	3.605	0.000
			3,5-Dichlorosalicylic acid	3.542	0.006
			4-Hydroxy-6-methyl-2-pyrone	3.214	0.000
			trans-Traumatic acid	2.882	0.000
		Depleted	sn-Glycero-3-phosphocholine	-4.953	0.000
			S-Adenosyl-homocysteine	-4.135	0.000
			D-(+)-Trehalose	-4.131	0.000
			2,3-Naphthalenedicarboxylic acid	-3.843	0.000
			Nicotinamide	-3.826	0.000
	Shared	Enriched	Ile-Leu	1.805	0.049
			Phe-Val	1.498	0.043
			Leu-Tyr	1.239	0.043
		Depleted	S-Adenosyl-homocysteine	-2.151	0.008
			D-(+)-Trehalose	-1.343	0.000
			Inosine	-1.136	0.049
*Microbacterium*	Control	Enriched	(.+/-.)-Camphor-10-sulfonic acid	5.156	0.000
			DL-Indole-3-lactic acid	5.012	0.008
			1,3-Cyclohexanedicarboxylic acid	4.567	0.002
			4-Hydroxy-6-methyl-2-pyrone.1	4.454	0.000
			2-((7-acetamido-1,2,3-trimethoxy-9-oxo-5,6,7,9-tetrahydrobenzo[a]heptalen-10-yl)amino)-N-(2,4-dimethoxyphenyl)-4-(methylthio)butanamide	4.168	0.000
		Depleted	sn-Glycero-3-phosphocholine	-5.023	0.000
			2,3-Naphthalenedicarboxylic acid	-4.549	0.000
			S-Adenosyl-homocysteine	-4.333	0.000
			D-(+)-Trehalose	-4.084	0.000
			Nicotinamide	-4.076	0.000
	Shared	Enriched	Urocanic acid	1.598	0.003
		Depleted	S-Adenosyl-homocysteine	-2.349	0.004
			Cytosine	-1.910	0.000
			Cytarabine	-1.850	0.000
			Crotonoside	-1.720	0.015
			2-O-Methyladenosine	-1.514	0.029
*R. qingshengii*	Control	Enriched	1,3-Cyclohexanedicarboxylic acid	4.533	0.000
			4-Hydroxy-6-methyl-2-pyrone.1	4.409	0.000
			2-Hydroxy-4-methylpentanoic acid	4.233	0.000
			2-((7-acetamido-1,2,3-trimethoxy-9-oxo-5,6,7,9-tetrahydrobenzo[a]heptalen-10-yl)amino)-N-(2,4-dimethoxyphenyl)-4-(methylthio)butanamide	4.200	0.000
			4-Hydroxyphenyllactic acid	3.636	0.006
		Depleted	sn-Glycero-3-phosphocholine	-5.194	0.000
			Nicotinamide	-4.246	0.000
			D-(+)-Trehalose	-3.871	0.000
			Val-Val	-3.448	0.000
			Val-Thr	-3.389	0.000
	Shared	Enriched	Crotonoside	1.009	0.013
		Depleted	3-Octanol	-1.120	0.007
			D-(+)-Trehalose	-1.084	0.000
*C. flaccumfaciens*	Control	Enriched	4-Hydroxy-6-methyl-2-pyrone.1	4.385	0.008
			2-((7-acetamido-1,2,3-trimethoxy-9-oxo-5,6,7,9-tetrahydrobenzo[a]heptalen-10-yl)amino)-N-(2,4-dimethoxyphenyl)-4-(methylthio)butanamide	4.205	0.003
			3,5-Dichlorosalicylic acid	3.486	0.009
			Monobutyl phthalate	3.228	0.009
			4-Hydroxy-6-methyl-2-pyrone	3.172	0.000
		Depleted	sn-Glycero-3-phosphocholine	-5.141	0.000
			Nicotinamide	-4.379	0.000
			Val-Val	-3.386	0.000
			D-Xylose	-3.239	0.000
			Val-Thr	-3.127	0.000

### CSC-A members utilize distinct methods for metabolizing urea degradation byproducts and mitigating stress, driven by *R. qingshingii* and *B. toyonensis*

Once we identified changes in gene expression and metabolite profiles during growth in urea-rich environments, we integrated our results to identify functional enrichment with multiple forms of evidence. For each species, we searched the KEGG reactome for reactions with at least one differentially enriched orthologous gene and one differentially enriched metabolite. These results were cross-referenced with the results of an expression-based KEGG pathway gene set enrichment analysis (GSEA) (Table S6). We identified 29 reactions supported by both transcriptomic and metabolomic data from *R. qingshengii*, 26 for *B. toyonensis*, 16 for *Microbacterium*, and 2 for *C. flaccumfaciens* (Table S7). *R. qingshengii*,* B. toyonensis*, and *Microbacterium* all shared decreased expressions of the succinate: quinone oxidoreductase reaction of the TCA cycle, leading to increased succinate.

In *R. qingshengii*, we observed 19 differentially expressed KEGG pathways via GSEA. The ABC transporters pathway exhibited positive enrichment (Table S6), with strong upregulation of the K11962, K11960, and K11961 orthologs coding for multiple subunits of the urea transport system, as well as the sulfur metabolism pathway, though no reactions with paired metabolite data were associated with this pathway. *R. qingshengii* also possessed the largest set of reactions with both transcriptomic and metabolic evidence, with multiple examples of nitrogen metabolism-related reactions acting on the major hub molecule L-glutamine, which was depleted during *R. qingshengii* growth in urea (Table S7). This resulted in significant repression of multiple glutamine-associated KEGG pathways such as the TCA cycle (ko00020) Biosynthesis of amino acids (ko01230), and Glyoxylate metabolism (ko01100). We observed upregulation of the dTDP-4-amino-4,6-dideoxy-D-galactose:2-oxoglutarate aminotransferase involved in producing nitrogen containing cell membrane components, and downregulation of multiple other reactions with glutamate as a reactant, such as (2 S)-2-amino-4-deoxychorismate: L-glutamate aminotransferase, which produces glutamine and chorismate from glutamate and (2 S)-2-amino-4-deoxychorismate. We also identified upregulation of 3-xanthine-dehydrogenase enzyme subunits mapping to the KEGG orthologies for *yagRST*, which bi-directionally converts xanthine to uric acid and xanthine to hypoxanthine alongside increased xanthine and hypoxanthine abundance during growth in urea. Similarly, GSEA analysis of *B. toyonensis* expression identified downregulation in the glyoxylate and dicarboxylate, and 2-oxocarboxylic acid metabolism KEGG pathways, which mapped to multiple related reactions with expression and metabolomic evidence. Of the 23 pathways differentially enriched in *B. toyonensis*, all were downregulated.

GSEA of *Microbacterium* expression identified 12 KEGG pathways, including enrichment of the two-component system pathway, increasing expression of regulator genes responsive to hypoxia and redox stress. *Microbacterium* also produced multiple reactions related to glutamate metabolism. These reactions include a decreased expression of the L-glutamate: ammonia ligase (ADP-forming) reaction which incorporates ammonia into glutamate, and increased expression of multiple transaminase reactions resulting in a depletion of the L-glutamine metabolite (Table S7). *C. flaccumfaciens* only yielded 2 reactions: decreased D-Xylose driven by the conversion of D-Xylose to D-Xylulose, and decreased expression of the proline utilization regulon repressor driving increased levels of proline. The only pathway enrichment detected for *C. flaccumfaciens* was significant depletion of the ABC transporter pathway.

**Fig. 8:**
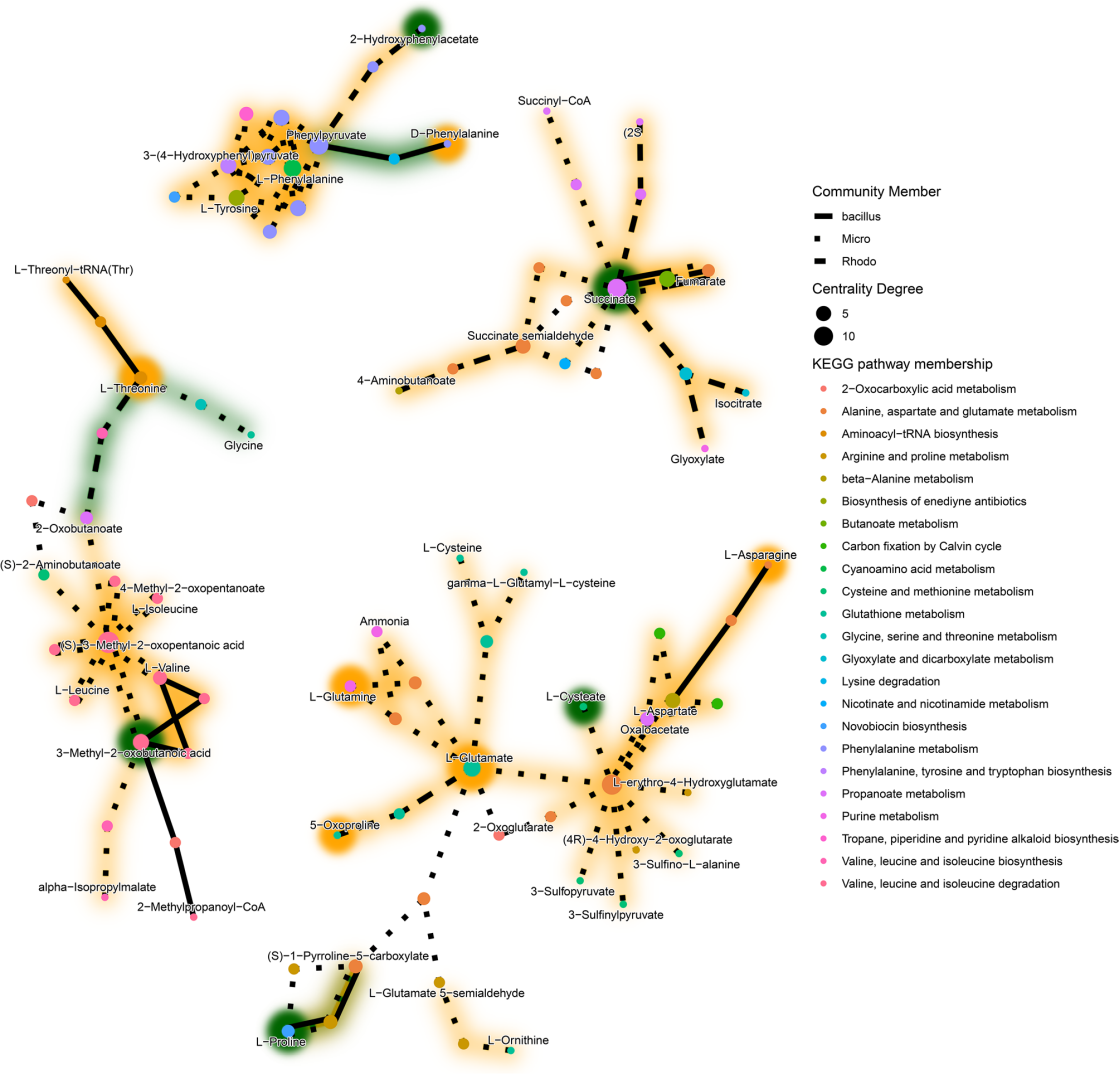
Community metabolite-reaction network of the dual evidence CSC-A reactome. Nodes represent KEGG compounds and edges represent KEGG reactions with a DE metabolite and at least one DE KEGG ortholog expression. Glow signifies DE, with green being enriched and orange being depleted. Node size indicates network degree. Nodes are colored by the KEGG pathway they are associated with and edge line type corresponds to the individual community member the evidence comes from

**Fig. 9:**
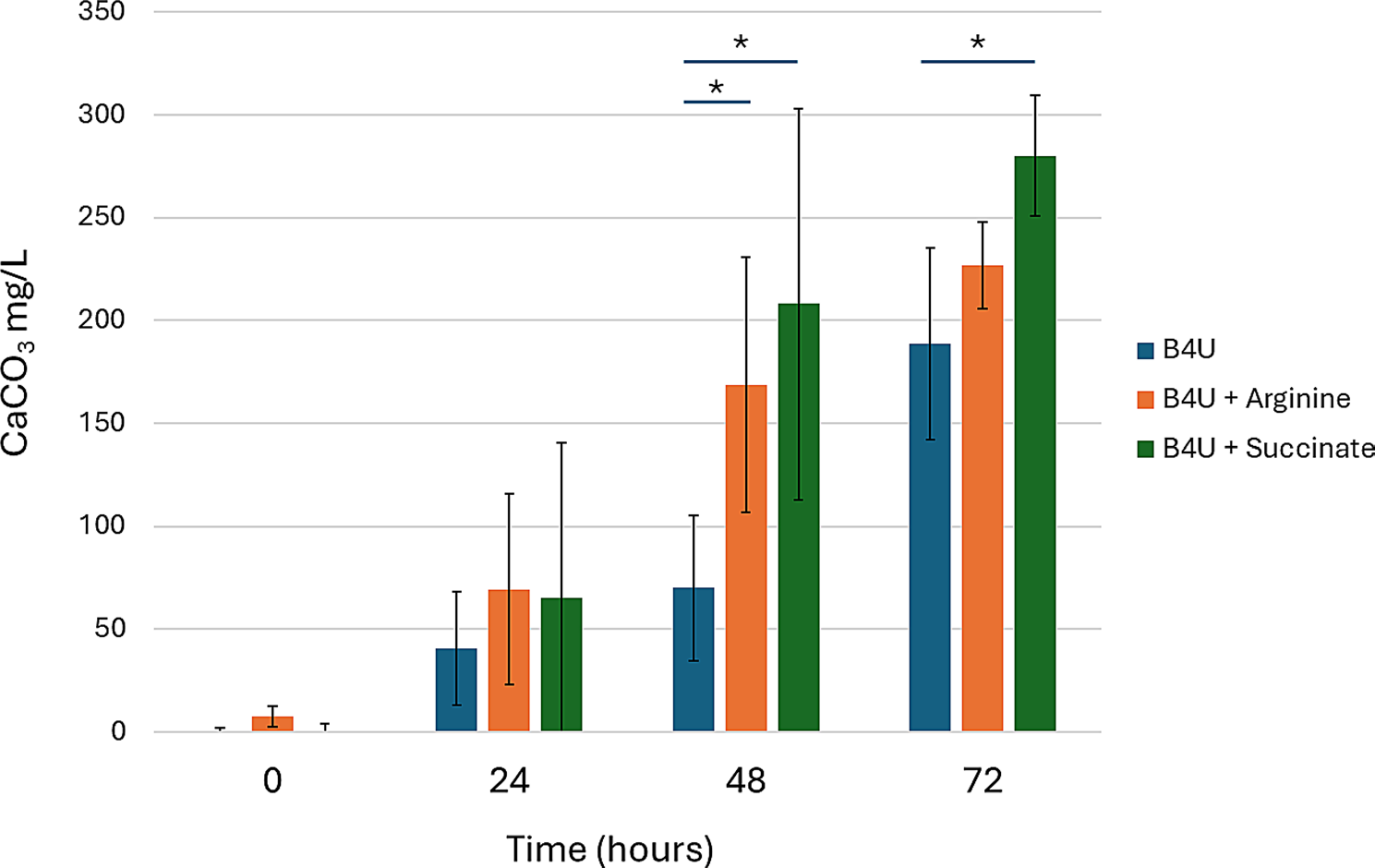
CaCO_3_ production with succinate or arginine added. Amount of CaCO_3_ is shown on the y-axis with time on the x-axis. Bars indicate growth of CSC-A in B4UZN media (blue), B4UZN media with arginine (orange) or B4UZN media with succinate (green). Error bars indicate standard deviation and a ‘*’ indicates a p-value of less than 0.05 compared to control media

After the analysis of each individual CSC-A member, we projected the entire set of community reactions as a DE metabolite-reaction network during growth under urea-rich conditions (Fig. 8). Two or more community members contributed to 4 subnetworks of the KEGG reaction network. Decreasing Phenylpyruvate (C00166) and increasing Succinate (C00042) compounds exhibited the largest network degree (10) and associated set of reactions, with a set or reactions shared among *R. qingshengii*, *B. toyonensis*, and *Microbacterium* connecting Succinate to Fumarate. L-Phenylalanine (C00079) and L-Glutamate (C00025) and a large set of reactions both decreased during growth in urea. Reactions and compounds associated with these subnetworks belonged to 23 amino acid, cofactor and vitamin, and carbohydrate metabolism KEGG pathways. Multiple amino acids such as L-proline (C00148), L-Cysteine (C00506), and Glycine (C00037) were all increased during growth in urea, though had low connectivity in the network. Due to the fact that we found succinate to be a major node in the network we next tested whether adding additional succinate to B4UZN media might increase the amount of CaCO3 precipitation. We also tested whether additional arginine may have an effect as we found it to be differentially abundant in our metabolomics analysis and arginine related pathways were found in our network. We found that the addition of arginine did lead to a 2.4-fold increase in carbonate production but this was only significant at one out of three tested timepoints (48 h after the start of the experiment) (Fig. 9). Succinate addition led to significantly increased carbonate production at both 48 (2.4-fold) and 72 h (1.48-fold) after the start of the experiment. This finding is a key link between our modeling data and laboratory production of CaCO_3_, showing that altering how we cultivate CSC-A based on model outputs can further enhance phenotypes of interest, increasing our ability to predict and control them.

## Discussion

Our study is an exploration of the synergistic metabolic exchange potentially influencing carbonate precipitation. We observed limited metabolic activity of one member (*C. flaccumfaciens*) consistent with an antagonistic interaction such as amensalism. Since CSC-A *C. flaccumfaciens* also contains the genetic components required for urea amidolyase-mediated urea degradation, it is possible *R. qingshengii* and *C. flaccumfaciens* are competing for urea. Less is known about the metabolic impacts of urea amidolyase in bacteria, which has been better characterized in fungi [36, 37]. However, the lack of an identified urea transporter in *C. flaccumfaciens* suggests that unless a cryptic transporter is present, urea must passively diffuse across the cell membrane. This is likely to occur slowly given the highly polar nature of urea, potentially limiting the impact of the urea amidolyase system on environmental levels of urea. This suggests that competition may drive most community dynamics in our experiments. More work is needed to fully distinguish the individual effects of both factors in urea degradation and community growth in CSC-A.

Our results here show that, even within a very small consortium, most species will sequester themselves into discrete niches with different roles in driving an overall community phenotype, here CaCO_3_ production. We found this to be the case for *R. qingshengii*, *B. toyonensis*, and *Microbacterium* during growth in urea but this observation is juxtaposed with *C. flaccumfaciens*, which had the lowest number of DEGs, a small impact on the community transcriptome, and a metabolome most like the shared reservoir. The relative sparsity of *C. flaccumfaciens* reactions mapped back to the transcriptome and metabolome also corroborates this. The life history trait analysis identified *C. flaccumfaciens* as having the longest MGT and highest OGT, which suggests that it may be outcompeted by faster growing community members whose OGT is more optimized for the temperature of lab experiments. *C. flaccumfaciens* are a widespread and “cosmopolitan” taxon, with large genomic potential for carbohydrate degradation, which we observe in our life history analysis [38]. *C. flaccumfaciens’* specialization in complex carbohydrate degradation may not be well optimized for in vitro lab experimentation with pre-digested, complete growth media. There was some evidence of increased co-expression of multiple sets of *C. flaccumfaciens* genes related to amino acid and central metabolism, potentially indicating the end of lag phase starting to occur at the 48-hour timepoint. Future studies in minimal media supplemented with complex carbohydrates, such as cellulose or chitin as they are naturally occurring sources in adjacent soil environments, could tease out environmental scenarios optimal for *C. flaccumfaciens* growth in CSC-A. Our prior work showed that, despite this species having little observable role in the phenotype of interest, there is no increase in CaCO_3_ production without *C. flaccumfaciens*. In fact, carbonate production levels drop. The observations in this study (lack of a clear role of *C. flaccumfaciens* in our multi-omic analysis) and our prior study speaks to the inclusion of species in a designed consortia even if they do not obviously contribute to the phenotype of interest. *C. flaccumfaciens* may become a more keystone member under alternative, more complex, carbon sources. Therefore, to design a consortium that may function under the widest range of conditions inclusion of additional species with non-overlapping potential is key, in CSC-A there is no negative effect seen with these additional member and potentially much larger gains.

Though the removal of *C. flaccumfaciens* seemed to minimally shift the functional niches of CSC-A members, we expect a more significant impact on the consortia in the absence of *R.qingshengii*. *R. qingshengii* was the only consortia member predicated to be capable of degrading and transporting urea inside the cell, so we expect it to act as a keystone species during growth in urea. As anticipated, the absence of *R. qingshengii* resulted in far more metabolic DEGs than removing *C. flaccumfasciencs*; a collective 9,009 DE versus 885, respectively. Unlike in the absence of *C. flaccumfaciens*, the number of DEGs increased at the 48-hour timepoint. This suggests a sustained metabolic effect on CSC-A, further supporting the hypothesis of niche reorganization occurring to compensate for the absence of *R. qingshengii*. This is also supported by the functions of some of the most upregulated genes in *B. toyonensis* in response to loss of *R. qingshengii*, such as CA and cyanate lyase, which we previously mentioned are alternative methods for precipitating calcium carbonate and producing ammonia [6, 12, 14]. While the possibility that these MICP-central enzymes are being activated was not explored in this experiment, upregulation of these enzyme-coding genes reinforces recent research efforts emphasizing their utilitarian potentials for enhancing MICP.

Another note of interest in the absence of *R. qingshengii* is the high expression of arginosuccinate synthase genes from *B. toyonensis.* When the full consortia was grown in urea-rich conditions, some arginine related reactions were favored—but the expression of arginosuccinate synthase genes is unique to conditions in the absence of *R. qingshengii*. These pathways may also be a factor in our observation that addition of succinate or arginine leads to increased CaCO3 production (at certain timepoints). Removal of *R. qingshengii* also resulted in significantly reduced expression of flagellar biosynthesis and protein synthesis genes from *Microbacterium.* This information suggests that in the absence of the CSC-A’s strongest urea degrading member, *B. toyonensis*’s ecological niche expands, taking over nitrogen metabolism and MICP activities, potentially at the cost of restructured arginine metabolism and *Microbacterium* metabolic activity. This expansion from realized niche breadth to fundamental niche breadth has also been observed in soil environments during lower biotic pressure [39]. However, it should be noted that without *R. qingshengii* there is little CaCO_3_ production so while *B. toyonensis* may move into this role CSC-A still loses its main phenotype of interest, a clear example that multi-omic analysis of how a community responds to changes in member abundance can sometimes be a poor predictor of community outcomes.

Aside from urea, other studies have determined that Ca^2+^ and dissolved organic carbon concentrations, environmental pH, and the availability of nucleation sites were all key factors influencing MICP [40]. *C. flaccumfaciens* and *Microbacterium* were both predicted to hold the largest complements of inorganic transport genes, which are important for regulating the concentration of Ca^2+^ in the cell at low levels while allowing for survival in high Ca^2+^ environments where MICP is likely to occur [1, 41–43]. Multiple reports suggest that Ca^2+^ transport is linked to active MICP, either by playing a significant role in mineralization or by promoting a localized increase in pH due to proton uptake, thus creating microenvironments conducive to MICP. Surprisingly, GSEA analysis of the transcriptome identifies significant repression of the Ca^2+^ pathway in *C. flaccumfaciens*, with simultaneous enrichment in *R. qingshengii*, a member not initially predicted to have as many complements to inorganic transport genes. This suggests that *R. qingshengii* may be adapting to the role in place of *C. flaccumfaciens*, influencing Ca^2+^ regulation. Thus, we hypothesize that certain members within the consortium are adapting to providing for Ca^2+^ regulation [42, 43].

MICP is a process underpinned by a complex network of interconnected gene expression and metabolite usages within a genetically diverse soil microbial consortium. We found that integrating the meta-transcriptomics and metabolomics data gave the most comprehensive view of metabolic integration during CSC-A growth in urea. The identified reactions confirm our prediction that metabolic flux through members of the community span multiple KEGG pathways and ecological niches—excluding *C. flaccumfaciens*, which had relatively metabolic response during CSC-A growth. *R. qingshengii* and *B. toyonensis* had large reaction sets, indicative of their important roles during urea degradation in CSC-A. Glutamate was central to one of the largest subnetworks of reactions, and cross-pathway glutamate metabolism were identified as highly modulated during growth in urea. Substantial evidence for metabolic activity in this glutamate subnetwork was contributed by *R. qingshengii*, *Microbacterium*, and *B. toyonensis*. *Microbacterium* showed increased expression of multiple aminotransferase reactions acting on or with glutamate, while *R. qingshengii* and *B. toyonensis* exhibited more heterogeneous expression of glutamate reactions. The temporal co-regulation analysis identified a group of genes largely from *R. qingshengii* with decreased expression of the de novo purine biosynthesis KEGG module which converts glutamine to IMP. This indicates that the metabolic activity involved in the glutamate network occurs mostly in the initial growth periods, when urea and the availability of other resources throughout the community are highest. This phenotype is particularly interesting given *Microbacterium* is not the primary urea degrader, nor is it capable of bicarbonate production via CA, so it should be expected to play a relatively small role in urea degradation. Though we did not see evidence of conversion of asparagine into ammonia for nitrogen, glutamine is the major, well-documented source of nitrogen for *Mycobacterium*, another Actinomycete, for tolerating pH induced stress [44]. Both glutamine and nitrogen molecules were depleted after growth, indicating that the ammonia created by MICP may not be available to *Microbacterium.* Other Actinomycetes have been shown to remodel TCA cycle metabolism in hypoxic environments, resulting in increased succinate and membrane potential [45], though we only find evidence of increased succinate in *R. qingshengii* profiles. Given the downregulation seen in Ile-Leu, Val-Thr, and other branched chain amino acids, it is likely that these mechanisms for reducing stress are taking place. *R qingshengii* uses a different set of reactions relating to glutamate and we find evidence of significant DE in oxoglutarate metabolism in the multi-evidence reaction network, primarily decreased in *R. qingshengii* and *B. toyonensis* and increased in *Microbacterium*. Thus, we find clear evidence of amino acids and other N containing compounds as well as glutamate is a hub compound for urea degradation in CSC-A, albeit through a complex, multi-pathway metabolic network.

We also found succinate to be an important node in the network, supported by multi-omic evidence across three species: *Microbacterium*,* B. toyonensis*, and *R. qingshengii.* The addition of this metabolite to B4UZN media led to significantly enhanced CaCO_3_ production. This represents a key link between our modeling work (based on multi-omic analysis) and the direct prediction and control of a phenotype of interest (carbonate production). Previous work linked growth on calcium succinate to altered TCA cycle flux, bypassing decarboxylation steps and reducing conventional metabolic CO_2_ [46] while still meeting energetic needs. In this scenario, MICP becomes dependent on CA to compensate, and indeed our results indicate CA is not only widespread among CSC-A members but is one of the largest positively DE genes upon community perturbation. CA acts as an efficient catalyst, increasing CO_2_ fixing by a factor of 10^6^ [47]. The resulting increase in HCO_3_^−^ likely increases carbonate alkalinity, promoting CaCO₃ supersaturation and precipitation [14]. CA mediated calcium carbonate precipitation occurs via a biochemically distinct pathway, independent of substrates; thus CSC-A has the ecological potential for functional synergism in complex environments external to our study. Further work is needed to determine the role of the other hub metabolites or reactions, as well as the impact of antagonism or competition between species, but this support of model predictions suggests that other modifications of the CSC-A growth environment, suggested by our network, could lead to further improvements in CaCO_3_ production.

This study, while comprehensive in an assessment of community level changes during urea degradation, did not thoroughly perturb or interrogate the community structure. This means we have not observed the effect of removing *B. toyonensis* or *Microbacterium* on community metabolic function. With the removal of *R. qingshengii*,* B. toyonensis* produced the largest change in transcription, suggesting it could overlap metabolically with much of the niche space that *R. qingshengii* currently utilizes. More phenotypic and genomic analysis is required to characterize the potentially novel *Microbacterium.* Depletion of amino acids, as was seen after growth in urea, has been suggested as a community strategy to modulate nitrogen metabolism to prevent competitors from accessing easy nitrogen sources [48]. This could be negatively impacting community MICP and growth in urea. Further work engineering the bacterial nitrogen regulatory system to reduce the ability to switch between nitrogen sources is needed. Finally, a more thorough exploration of metabolic potential could be conducted by more phenotypic testing, such as carbon source growth assays or computational modeling techniques such as genome scale modeling, to gain a better understanding of how community dynamics and competition result in growth and production of metabolites. The purpose of this study was to characterize the genetic, metabolic, and functional potential of a previously identified community of organisms capable of using urease mechanisms to generate calcium carbonate. A logical next step would be the exploration of how this work could be applied to in situ calcium carbonate precipitation. Natural soil microbiomes contain many more species and potential interactions than we use here but each of the species that are a part of CSC-A are also found in the soil of Davis, CA. To promote in situ calcite formation at this site we could directly apply CSC-A to the soil as an amendment strategy. This could be done in conjunction with urea application in the form of fertilizer which may have a two-fold benefit: excess nitrogen from urea is converted into biomass, reducing fertilizer runoff and calcium carbonate is formed, locking carbon into stable inorganic forms in soil. An alternative approach would be to take the knowledge we have gained and apply this to soil without a microbial component. We show here a direct strategy: succinate application, that improves carbonate formation, adding succinate directly to soil, a prebiotic approach, is worth exploring for a similar effect. A metagenomic and metatranscriptomic analysis of the soil would also provide data on which species contain and express urease enzymes, allowing us to target our approach to particular soil types.

## Conclusions

Our study provided an analysis of community level changes during urea degradation, finding that CSC-A members have a diverse suite of metabolic potential, and phenotypic evidence of niche specialization in stress response and metabolism, as well as generalization in key metabolic functions such as denitrification. Exclusion experiments of two individual species of CSC-A emphasized the identification of a keystone species, *R. qingshengii* along with species niche specialization that may reorganize under specific sets of time-sensitive conditions, such as accessibility to urea in the environment. Exclusion and subsequent analysis of the transcriptome of another member, *C. flaccumfaciens*, revealed that inclusion of species with no phenotypically observable roles is still crucial when designing consortia to mitigate the effects of growth conditions or competition. We also show that our predictions of CSC-A interactions can be used to perturb the system in the laboratory for enhanced expression of a phenotype of interest. Multiomic integration of our datastreams resulted in the identification of 29 different reactions across 4 subnetworks of a KEGG reaction network. The identification of reactions including oxoglutarate, succinate quinone reducatases, and other amino acids and vitamins critical for glutamate and succinate metabolism, evidence suggests that these two compounds serve as major hubs in our complex, multi-pathway metabolic consortium network. Among the metabolites predicted to be integral to CaCO_3_ precipitation, only the addition of succinate to our growth media led to an average increase of two-fold more CaCO_3_ produced throughout the duration of the experiment when compared to the yield of CaCO_3_ during growth in unenriched media—a culminative result of empirical validation of our modeling work. There is still much that is unknown about the mechanistic driving forces behind CSC-A-enabled MICP and its scalable application in the field, but this study highlights the strength of insights that can be gained from integrating multiple streams of ‘omics analyses. This foundational work allows insight for future work engineering new niche restrictions or enhancing calcium carbonate precipitation to increase the efficiency and productivity of this novel biosystem.

## Supplementary Information

Below is the link to the electronic supplementary material.

Supplementary Material 1: Supplementary Figure 1. Heatmap of KEGG metabolite enrichment and depletion in each community well compared to the shared reservoir. Cell color represents differential abundance, with red indicating metabolite enrichment and blue indicating metabolite depletion.

Supplementary Material 2: Supplementary Table 1. Optimum generation time, optimum temperature, and genome size as found in literature reviews as well as through genomic reconstruction. Supplementary Table 2. Complete table of gene expression given different CSC-A communities (without Curtobacterium vs. without Rhodococcus) when grown in urea-rich media, inclusive of genes unable to be mapped to the KEGG ortholog database. Supplementary Table 3. Global gene expression levels, normalized with DESeq2. Supplementary Table 4. Metadata information for RNA-seq data. Supplementary Table 5. Global metabolite abundance levels. Supplementary Table 6. GSEA results for transcriptome of CSC-A when grown in urea. Supplementary Table 7. Reactions predicted from multi-omic analysis.

## Data Availability

Genomes for the four species that make up CSC-A are deposited in NCBI with the following Biosample accession numbers: SAMN55276877 (Rhodococcus qingshengii), SAMN55276878 (Curtobacterium flaccumfaciens), SAMN55276879 (Microbacterium sp.) and SAMN55276880 (Bacillus toyonensis). RNA-seq data has been deposited in the Gene Expression Omnibus (GEO) with accession number GSE320329. Metabolomics data has been deposited in MassIVE with accession number MSV000100667.
