# Supplementary figures and images for: Multi-omic characterization of a soil microbial consortium reveals critical role of succinate and glutamate metabolism during calcium carbonate precipitation
Source: BMC Biotechnol. 2026 Jun 23;26:80. doi: 10.1186/s12896-026-01136-y (PMC13292334; doi:10.1186/s12896-026-01136-y)

## Slide 1
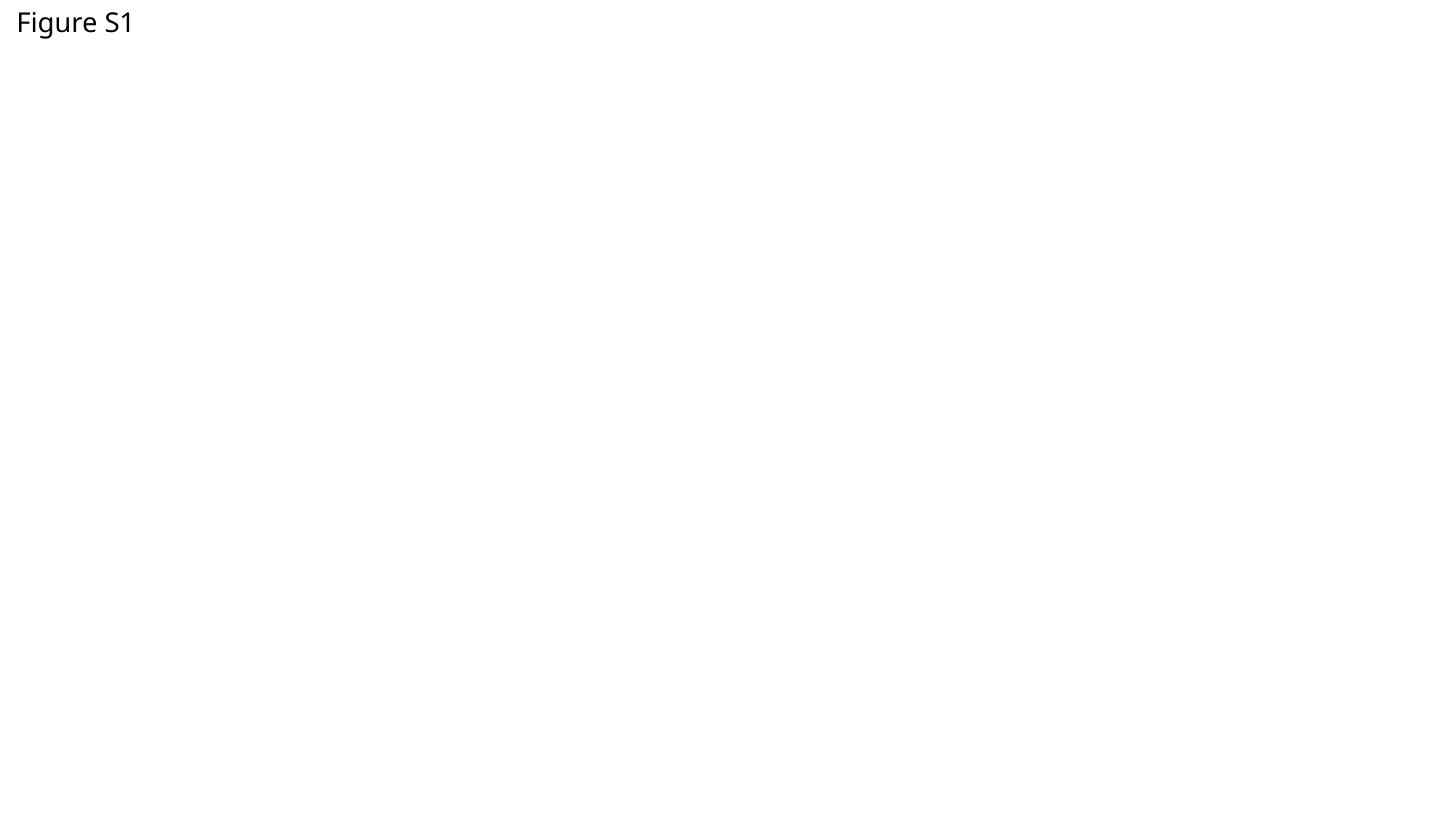

Figure S1

Supplement: Supplementary file 1 — Supplementary Material 1: Supplementary Figure 1. Heatmap of KEGG metabolite enrichment and depletion in each community well compared to the shared reservoir. Cell color represents differential abundance, with red indicating metabolite enrichment and blue indicating metabolite depletion. [file 12896_2026_1136_MOESM1_ESM.pptx]
